# Canine babesiosis – a disease rarely considered in the context of male infertility

**DOI:** 10.1186/s13620-020-00174-y

**Published:** 2020-11-06

**Authors:** Anna Domosławska, Sławomir Zdunczyk

**Affiliations:** Department of Animal Reproduction with Clinic, University of Warmia and Mazury in Olsztyn, Olsztyn, Poland

**Keywords:** *Babesia canis*, Fertility problems, Semen quality, Dogs

## Abstract

**Background:**

Little is known about the impact of babesiosis on semen quality and fertility in dogs.

**Case presentation:**

Four cases of infertility in male dogs after infection with *Babesia canis* are described. In all dogs sperm quality was low. Two dogs were castrated pharmacologically or surgically. In two dogs fertility was restored after supplementation with selenium and Vitamin E. As possible causes of spermatogenesis disorders due to the treatment of infection with *Babesia canis* with imidocarb, fever and disturbed testicular microcirculation are discussed.

**Conclusions:**

These cases indicate that if males have fertility problems, question about babesiosis infection in the past should be a permanent point in the clinical interview.

## Background

Ticks are well designed to transmit disease agents such as viruses, bacteria and protozoa that can cause conditions such as babesiosis, ehrlichiosis, hepatozoonosis, rickettsiosis and others [1]. In Poland, the most common tick-borne disease in dogs is Babesiosis caused by the intraerythrocytic protozoan parasite *Babesia canis* [2]. The clinical picture of the signs varies, from subclinical infections to multi-organ failure, with a risk of death. The clinical signs are progressive haemolytic anaemia, fever, apathy, pale mucous membranes and dark urine [1–3]. Little or nothing has been said about the impact of babesiosis on breeding dogs in terms of their reproduction [4]. So far, there are no studies on the influence of babesiosis on the quality of semen in dogs.

## Case presentation

Four stud dogs were presented for semen evaluation to the Department of Animal Reproduction with Clinic because of acquired infertility, defined as conception failure in at least 3 matings with different females. They were: a 4 year-old Collie rough (I), 4 year-old Alaskan malamute (II), 4 year-old Beauceron (III) and 3.5 year-old Cavalier King Charles spaniel (IV). All dogs had a history of infection with babesiosis during the previous 24 months. Babesiosis was diagnosed by light microscopy evaluation of blood smears for parasite visualization. The infection proceeded with a significantly increased internal body temperature (39.0–40.5 °C) lasting from 1 to 3 days. Males I and IV had had the infection twice in the previous 6 months. The shortest time from the last infection to the first semen analysis was 3 months. The animals had a history with the following treatment during the infection: imidocarb diproprionate (6 mg/kg b.w. *s.c.* once, Imizol, MSD Animal Health), enrofloxacin (5 mg/kg b.w. *p.o.* daily for 7 days), and tolfenamic acid (4 mg/kg b.w. s.c. twice in 24 h interval, Tolfedine 4%, Vetoquinol). The dogs were subjected to general clinical examination including inspection and palpation of genital organs, ultrasound scanning of the prostatic gland, testes and epididymis (Esoate MyLab 30 Vet Gold, Genova, Italy, equipped with a microconvex probe, 6.6–8.0 MHz) and semen collection. Semen was collected by manual manipulation as described by Linde-Forsberg [5] in the presence of a teaser bitch in heat. The ejaculates were collected into pre-warmed (36–38 °C) glass tubes as sperm fraction and prostate gland fraction separately. The semen was evaluated immediately after collection. Volume, concentration and motility parameters: percentage of motile spermatozoa (MOT), percentage of spermatozoa with progressive motility (PMOT), velocity average pathway (VAP), velocity straight line (VSL), velocity curvilinear (VCL), amplitude lateral head (ALH), beat cross frequency (BCF), straightness (STR), linearity (LIN), and RAPID, MEDIUM, SLOW, and STATIC motility subcategories were assessed by computer assisted sperm analysis (CASA) using a Hamilton Thorne sperm analyser (IVOS 12.3). The percentage of live and dead spermatozoa was estimated on dried smears stained with eosin/nigrosin. At least 200 spermatozoa were assessed using microscopy. Unstained spermatozoa (intact membrane) were classified as live and red stained spermatozoa (defect membrane) were classified as dead. For the assessment of sperm morphology monochromatic Diff-Quick stain was used. Morphological deviations in the spermatozooa were evaluated as acrosome, head defects, tail defects and proximal cytoplasmatic droplets [6].

Two hundred spermatozoa were evaluated per slide, representing 100%. Reference values for semen quality parameters were taken from Günzel-Apel et al. [7], Rijsselaere et al. [8] and Kirchhoff et al. [6] and are given in Table 1. The samples of second and third fraction of ejaculate were examined bacteriologically in the routine manner.

**Table 1:** Semen parameters in dogs, which were infected (after having been infected) with Babesia canis at 1st presentation and after supplementation with selenium and Vitamin E

Parameter	Male I		Male II	Male III	Male IV		Values in fertile males
	at 1st presentation	after supplementation	at 1st presentation	at 1st presentation	at 1st presentation	after supplementation	
Volume (ml)	2.0	3.5	2.5	2.0	0.8	1.5	0,5–2.0^a^
Concentration (×10^6^/ml)	35.5	308	450	34	59.5	178	292.6 ± 208.3^b^
MOT (%)	4	64.0	80.0	5.0	78.0	72.0	88.3 ± 18.4^b^
PMOT (%)	1	29	68	0	7	54	60–70^a^
VAP (μm/s)	64.6	127.3	157.4	53.5	86.9	142.9	124.3 ± 19.7^b^
VSL (μm/s)	46.2	122.4	132.2	36.4	58.6	127.5	113.0 ± 20.2^b^
VCL (μm/s)	159.0	180.2	212.0	143.9	193.2	222.7	160.7 ± 19.7^b^
ALH (μm)	4.3	6.1	6.4	0.0	8.6	7.7	5.0 ± 0.7^b^
BCF (Hz)	36.2	35.4	32.1	34.9	22.7	38.8	26.2 ± 4.4^b^
STR (%)	70	96	82	69	67	88	88.9 ± 3.4^b^
LIN (%)	29	69	62	26	30	57	70.1 ± 7.5^b^
RAPID (%)	2	39	82	1	15	60	65.2 ± 21.7 ^b^
MEDIUM (%)	21	31	10	21	63	12	3.4 ± 2.4^b^
SLOW (%)	4	18	7	5	8	19	19.7 ± 12.8^b^
STATIC (%)	73	12	0	73	15	9	11.8 ± 14.4^b^
Morphological abnormal sperm (%)	89	52	90	60	58	35	10–25^a^
Acrosomal defects (%)	7	5	7	6	3	3	2.3 ± 1.0^c^
Head defects (%)	29	18	39	23	15	9	4.0 ± 3.0^c^
Tail defects (%)	11	7	4	6	4	5	12.3 ± 4.6^c^
Proximal cytoplasmic droplets (%)	42	22	40	25	38	18	10.3 ± 12.1^c^
Live (%)	38	65	87	20	61	79	90–95^a^

^a^reference values according to Günzel-Apel et al. [7]

^b^Rijsselaere et al. [8]

^c^Kirchoff et al. [6]

Results of the semen evaluation are shown in Table 1. In all dogs sperm quality parameters were poorer than the values reported for fertile dogs [6–9]. In males I, III and IV the sperm concentration was below the reference value of 300 × 10^6^/ml, and the percentages of progressively motile spem were very low (1.0, 0.0 and 7.0%, respectively) compared to the reference value of 60–70%. Also, the other CASA motility parameters in dogs I, III, and IV were lower compared to values reported for fertile dogs. There was also a significant decrease in the viability of spermatozoa in these three males. In male II these above-mentioned parameters were within the reference range. The percentage of morphologically abnormal sperm was high in all dogs and ranged from 35 to 90%, while the reference value is 10–25%. The predominant sperm defects were head defects and proximal cytoplasmatic droplets (15–39% and 38–42%, respectively). The percentages of live spermatozoa were low in males I, III and IV (20–61%).

To improve semen quality the males were subjected to oral supplementation with selenium (6 μg/kg organic selenium from yeast) and Vitamin E (5 mg/kg) for 60 days (Semevet, VetExpert®). After this time, semen evaluation was repeated in males I and IV. Results of the semen evaluation in two males after 60 days of Se and Vit E supplementation are shown in Table 1. An improvement in volume, concentration and motility - especially progressive movement, sperm morphology and vitality - was observed in both males. Further antioxidant supplementation was recommended to maintain the sperm enhancement effect.

The owners of the other 2 males did not proceed with the treatment. Male number II mated only once after 2 months from the begining of the supplementation but the female did not become pregnant and the male was castrated pharmacologically (Suprelorin, Virbac). Male III was subjected to surgical castration before the 60 days of supplementation ended. However, histopathological examination of the testicles was not possible since the castration took place in another veterinary clinic and the dog owner informed us about this afterwards.

Male I mated naturally with four bitches within 7 weeks after 2 months from the end of Se and Vitamin E supplementation. Three of these had a live multiple pregnancy. One developed endometritis diagnosed 3 weeks after mating by ultrasonography and was subjected to ovariohysterectomy. Male IV was mated with two females by artificial insemination within 10 weeks after 2 months from the end of Se and Vitamin E supplementation, and both confirmed pregnancies diagnosed 25 days after insemination by ultrasonography.

## Discussion and conclusions

All dogs had a low quality of semen during the first sperm evaluation. The causes of spermatogenesis disorders may have been a complex process of many overlapping factors, but in these cases the effect of inflammation leading to an elevated body temperature or microcirculation disorders in the testicles during babesiosis can be suspected. An imidazole-induced spermatogenesis disorder is unlikely since there are no data on adverse effects of imidazole on semen quality [10, 11].

In dogs, as in other mammalian species with extra-corporal testes, testicular function is dependent on intra-testicular temperature, which is 2.5–5.3 °C below body temperature [12]. In the human there is evidence of a link between elevated intra-testicular temperature and azoo- or oligozoospermia, impaired motility and morphology [13]. In mice, short-term heating of the scrotum leads to disruption of spermatogenesis, a reduced number of testicular haploid cells and decreased testicular weight [14]. The effect of mild, short-term scrotal hyperthermia on semen parameters in dogs was investigated by Henning et al. [12]. A single increase in scrotal temperature by 3 °C for 48 h did not cause substantial changes in sperm quantity and quality. After a second scrotal insulation after 9 weeks the total sperm count and the amount of morphologically intact spermatozoa only tended to decrease. Thus, in contrast to other species, canine testes are less susceptible to hyperthermia. However, a high rise in body temperature during babesiosis cannot be excluded as a cause of spermatogenesis disorders. Hyperthermia induces a higher demand for oxygen supply in the testes tissue, which might be not compensated for, since vasodilatation of the testicular artery in the pampiniform plexus is an insufficient mechanism for increasing of blood flow in the testis [15]. Hypoxia promotes the increase of recative oxygen species leading to oxidative stress and disturbed spermatogenesis [16]*.*

On the other hand, it must be remembered that the damage to the host is more the consequence of the host’s immune response to the infection, than direct damage by the parasite [17]. During babesiosis a cytokine storm is observed, which is a response to multiple organ damage, including the testicles. Enhanced production of cytokines and chemokines (ie, IL-6, IL-8, TNF-alpha) can trigger oxidative stress [18–20]. This phenomenon involves complex and multiple interactions among immune and germ cells, resulting in the alteration of spermatogenesis [21].

In cases where the animal died as a result of infection with *Babesia spp*., histopathological examination of the testicles confirmed damage to the tissue in the form of necrosis and numerous vessels displayed segmental inflammatory changes [4]. Segmental degeneration of the seminiferous tubules was also found, characterized by the presence of multinucleated cells in their lumen. The mechanism of testicular tissue damage in the course of this disease is still unclear, although the observed haemolytic anaemia may generate testicular microcirculatory disruption and blood flow reduction, together with oxidative stress.

Our report clearly demonstrated that dogs suffering from babesiosis show poor semen parameters, especially motility. The high percentage of spermatozoa with head defects indicates disturbed spermatogenesis in the testes. The high percentage of spermatozoa with proximal cytoplasmatic droplets and low motility parameters indicate damage to the epididymites, in which spermatozoa acquire motility and membrane integrity [22]. The variation in sperm quality parameters between dogs may be due to individual differences in the response to infection with Babesia canis.

In our cases, the owners refused testicular biopsy, which could have confirmed or excluded tissue degeneration and would perhaps have lead to other conclusions and results. Although the semen quality was beyond the physiological range in males I and IV, pregnancies were obtained. In male IV there was a surprisingly significant increase in progressive motility and the RAPID cell sperm subpopulation. These improvements resulted in confirmed pregnancies.

Supplementation with antioxidants can lead to sperm improvement, as was demonstrated in a previous report [23]. In the cases described, supplementation with selenium and Vitamin E as antioxidants also improved the quality of semen and restored fertility in two dogs. It must also be stressed that the mated females were the same ones which had previously mated unsuccessfully.

This case report indicates that babesiosis can cause infertility in dogs. A question about the possible occurrence of babesiosis should always be included in interviews with the owners of stud dogs, especially in tick endemic areas.

## Data Availability

All data generated or analysed during this study are included in this published article.
